# The role of the expanded food and nutrition education program in improving healthy eating index scores for low-income households in selected counties in Texas

**DOI:** 10.1371/journal.pone.0320607

**Published:** 2025-05-02

**Authors:** Oral Capps, Xingguo Wang

**Affiliations:** Department of Agricultural Economics, Texas A&M University, College Station, Texas, United States of America; GLA University, INDIA

## Abstract

The Expanded Food and Nutrition Education Program (EFNEP) is a federal initiative aimed at improving the dietary behaviors and nutrition knowledge of low-income households. This study evaluates the impact of Texas EFNEP on the dietary quality of participants using data from across ten counties over four fiscal years (2019–2022). Dietary quality was assessed using the Healthy Eating Index-2015 (HEI), calculated from 24-hour dietary recalls collected before and after participation in the program. The study analyzed changes of HEI scores across fiscal years, counties, socio-demographic characteristics, and public assistance program participation. The Texas EFNEP intervention resulted in a statistically significant improvement in overall HEI scores, 4.23 on average. The greatest dietary improvements were noted in Tarrant and Hidalgo counties. Among racial groups, participants identified as Asian showed the most improvement on average, followed by participants identified as white and as black. On average, Hispanic participants experienced greater dietary improvements than non-Hispanic participants. Based on regression analysis, geographic location and participation in public assistance programs such as the Child Nutrition Program (CNP) significantly impacted total HEI scores, but age, income, and hours taught in EFNEP were not statistically significant determinants. Statistically significant improvements were detected in eight of the nine adequacy components of the HEI, including total fruit, whole grains, and dairy. Concerning the moderation components, statistically significant changes were evident for refined grains, added sugar, and saturated fat. However, the program was less effective in moderating sodium intake, a known dietary challenge in low-income populations. The findings suggest that the Texas EFNEP contributed to improvements in overall dietary quality, including enhancements in both adequacy and moderation components of the Healthy Eating Index. These findings are consistent with prior research concerning the effectiveness of EFNEP studied in other states and regions.

## Introduction

Nutrition and food security are national health concerns, especially among low-income populations that disproportionately experience poor health. The Expanded Food and Nutrition Education Program (EFNEP) is a federal nutrition education program funded by the United States Department of Agriculture National Institute of Food and Agriculture (USDA-NIFA) that targets low-income families in all states and the District of Columbia as well as six U.S. territories (American Samoa, Guam, Micronesia, Northern Marianas, Puerto Rico, and the Virgin Islands). EFNEP began in 1968 with the goal of alleviating the link between poverty and nutrition [1]. The program’s goals are to “assist low-income audiences in acquiring the knowledge, skills, attitudes, and changed behavior necessary for nutritionally sound diets, and to contribute to their personal development and the improvement of the total family diet and nutritional well-being” [2].

Past studies pertaining to state, regional, and national evaluations have shown that EFNEP is not only effective in bringing about changes in nutrition-related behaviors but also in changing diet quality [1,3–17]. Evaluations of similar programs to EFNEP, such as of SNAP-ed programs on diet quality, reveal that education alone is often insufficient to change dietary intake and diet quality to a meaningful degree [18].

According to Atoloye et al. [19], in recent years, emphasis has been placed on dietary quality indices, namely the Healthy Eating Index (HEI), as a measure of how well food and nutrient intakes of participants aligned with the Dietary Guidelines for Americans [14,16,17,20]. Guenther and Luick [14] conducted a secondary analysis of dietary data collected from EFNEP participants in eight states of the US Census Mountain region (Arizona, Colorado, Idaho, Montana, Nevada, New Mexico, Utah, and Wyoming) from October 2010 to September 2011. Statistical analyses were conducted based on entry and exit total and component HEI 2005 scores. Age, race/ethnicity, pregnancy/lactation status, education level, and state were considered in their sample. However, no analyses of the respective HEI 2005 component and total scores were conducted by socio-demographic characteristics.

Weatherspoon et al. [16] conducted a regression analysis of HEI scores for Michigan based on the 2005 Dietary Guidelines for fiscal years 2011 and 2012. The dependent variable corresponded to the level of the HEI score. Explanatory variables considered were household income, location of residence, educational attainment, age, race, and ethnicity; number of lessons received; and seven behavioral variables related to planning meals before grocery shopping; comparing prices when shopping; running out of food before the end of the month; using a grocery list when shopping for food; thinking about healthy food choices; using the information presented on food labels; and budgeting enough money for food.

Perkins et al. [17] considered changes in the HEI as well as changes in 13 components of the HEI for Maine based on the 2005 Dietary Guidelines for fiscal years 2013–2016. Statistically significant differences were conducted concerning changes in the HEI as well as changes in 13 components of the HEI among household income, location of residence, educational attainment, age, race, ethnicity, number of hours taught, and participation status in various food assistance programs.

Finally, Gillis et al. [20] conducted a secondary analysis of information collected from EFNEP participants across the United States from October 2012 to September 2014 to test overall and component changes in HEI scores based on the 2005 Dietary Guidelines. Demographic characteristics included age, education, gender, and race/ethnicity.

Texas is the largest EFNEP program, but to date no studies have been published dealing with the impacts of the Texas EFNEP regarding ***nutritional status and dietary improvement***. Valdez [21] conducted a follow-up study of the small group teaching approach used in the Texas EFNEP. Cullen et al. [22] documented the ***potential*** public health impact that the Texas EFNEP could have on dietary behaviors for at-risk populations and ultimately obesity prevention efforts. Cullen et al. [23] examined the relationships between participant goal attainment and changes in mediating variables and food choice outcomes from a modified curriculum for the Texas EFNEP promoting healthy home food environments and parenting skills related to obesity prevention.

This research constitutes a single-state, case-study approach and fills this research void. Notably, our study makes unique contributions to the extant literature. Aside from the exclusive focus on Texas, our study considers total and component HEI scores based on the 2015 Dietary Guidelines. Previous studies relied on HEI scores derived from the 2005 Dietary Guidelines. Additionally, we center attention on changes in overall HEI scores by socio-demographic characteristic as well as by participation status in public assistance programs. Further, unlike the previously mentioned studies, we conduct rigorous statistical analyses conditional on non-parametric tests as well as a regression analysis using weighted least squares conditional on a detailed list of explanatory variables. Moreover, our data analysis corresponds to coverage from October 2018 to September 2022. In this way, we consider not only the impact of the pandemic but also the impact of changes in the U.S economy, notably inflation. Though limited to Texas, understanding the effectiveness of EFNEP is important to stakeholders, particularly considering state budget considerations.

The remainder of the paper is structured as follows. In the next section, we provide the objective and hypotheses. Subsequently, we center attention on the target population, sample, and setting. Then we focus on the data analysis associated with the change of the total HEI score before and after the EFNEP intervention. In this section, we explicitly consider the change of the total HEI score by fiscal year, county, race, and participation status in various public assistance programs. Thereafter, we present a regression analysis to explore the relationships among various factors concerning their impact on the change of the total HEI score. To conclude, we discuss the empirical results, and we provide the limitations and implications for research and practice.

## Objective and hypotheses

The overarching objective was to examine the impact of the Texas EFNEP in improving Healthy Eating Index (HEI) scores for low-income households located in selected counties in Texas. The foundation of this study rests on the use of pre/post-secondary data analysis gathered from the national EFNEP database concerning adults enrolled in the Texas EFNEP over four fiscal years from 2018 to 2022. This four-year period allows the assessment of changes in nutritional status before and after COVID-19 as well as the assessment of the rise in inflation more recently. Because of the reliance on secondary data, this study was deemed exempt by the Texas A&M University Institutional Review Board.

Initially, we calculated the change of HEI scores based on a 24-hour dietary recall for each participant. In addition, we considered socio-demographic factors (age, race, household size, household income, number of children, gender, ethnicity, education level, rural/urban residence), participation in various food assistance programs and the number of hours taught. Our hypotheses are succinctly summarized as follows: (1) diet quality of adults as measured by the change of HEI increases because of the Texas EFNEP; (2) the magnitude of the change of HEI is positively related to the total number of hours taught in the Texas EFNEP; (3) the change of HEI differs by county and by fiscal year in Texas; (4) the change of HEI is higher for individuals that participate in food assistance programs compared to individuals who do not participate in these programs; (5) the change of HEI is not uniform across food assistance programs; and (6) the change of HEI varies across socio-demographic factors in Texas.

### Target population, sample, and setting

Texas EFNEP targets low-income families with young children that are at high risk for poor nutrition and food insecurity. The program assists them in acquiring the knowledge, skills, and attitudes necessary for nutritionally sound diets and for managing their food resources. Survey data were collected over four consecutive fiscal years (2018/2019, 2019/2020, 2020/2021, and 2021/2022) from 5,855 adult individuals enrolled in EFNEP across ten diverse counties in Texas: Bexar, Cameron, Dallas, El Paso, Harris, Hidalgo, Kleberg, Nueces, Tarrant, and Travis. The fiscal year is defined as the 12-month period beginning on October 1 of the previous year and ending on September 30 of the following year. Five individuals from Willacy County were surveyed, but due to this insufficient number for statistical analysis, these individuals were excluded. Each participant was assigned a **unique survey ID each year**. The same ID was not present in multiple years. Consequently, the database for analysis corresponds to repeated cross-sections in lieu of an unbalanced panel.

In each fiscal year, individuals included in the study were required to complete two 24-hour dietary recalls, one before participating in EFNEP and the other after completing the program. Each recall was interviewer-administered by trained EFNEP personnel from Texas A&M AgriLife Extension Service following a standardized protocol to ensure consistency and accuracy. The 24-hour food recall has been used to monitor the dietary intake of EFNEP participants since the program’s inception. Various supplementary questionnaires also were used to measure changes in participants’ knowledge, skills, practices, and behaviors.

Based on this information, the Healthy Eating Index-2015 (HEI) total score for each participant was calculated, which measures adherence to the Dietary Guidelines for Americans 2015-2020 (DGA) [24]. At the time of data collection, the HEI-2015 was the standard. Although the HEI-2015 now has been replaced with the HEI-2020, for adults, there is no difference between the HEI-2015 and the HEI-2020. The Web-Based Nutrition Education Evaluation and Reporting System (WebNEERS) uses information from various databases concerning food recalls to calculate HEI scores. The underlying database sources are a combination of the Food Patterns Equivalents Database (FPED) and the Food and Nutrient Database for Dietary Studies (FNDDS). Moreover, the HEI calculations were based on the population ratio method as opposed to the Markov Chain Monte Carlo (MCMC) method.

The HEI total score ranges from 0 to 100, with higher scores indicating closer adherence to DGA recommendations. The score is derived as the sum of scores associated with 13 components that assess diet quality—9 adequacy components and 4 moderation components. Adequacy components relate to the intakes of total fruit, whole fruit, total vegetables, greens and beans, whole grains, dairy, total protein foods, seafood and plant proteins, and fatty acids. Higher adequacy scores indicate higher ratios of the intakes of these food groups relative to their calorie intakes, except for fatty acids. A higher score of fatty acids represents a higher intake of this food component. Moderation components relate to the intakes of refined grains, sodium, added sugars, and saturated fats. Lower intakes of these elements result in higher moderation scores indicate higher ratios of the intakes of these elements relative to their calorie intakes, except for added sugar and saturated fats. The primary variable of interest in this study is the change of the HEI total score before and after the EFNEP intervention. Simply put, the change in HEI serves as a proxy for dietary improvement in this study.

Additionally, in the post-survey, the total number of hours participants were taught in EFNEP were recorded and collected. Also, participants were asked to provide demographic information, including age, gender, race, ethnicity, pregnancy status, and nursing status as well as household characteristics such as household size, number of children, household income, and location of residence (rural/urban). In the Texas EFNEP, the education level of participants was not considered. Consequently, we are not able to address differences in HEI total scores by education level.

Further, participation status in other public assistance programs were solicited. Individuals were allowed to select multiple programs. The response options included: (1) Child Nutrition Programs (CNP), (2) the Supplemental Nutrition Assistance Program (SNAP), (3) the Food Distribution Program on Indian Reservations (FDPIR), (4) Head Start, (5) Temporary Assistance for Needy Families (TANF), (6) the Emergency Food Assistance Program (TEFAP), and (7) the Special Supplemental Nutrition Program for Women, Infants, and Children (WIC) or Commodity Supplemental Food Program (CSFP). Child Nutrition Programs (CNP) consist of five individual programs aiming to help ensure that children receive nutritious meals and snacks that promote their health and educational readiness, including the National School Lunch Program (NSLP), the School Breakfast Program (SBP), the Child and Adult Care Food Program (CACFP), the Summer Food Service Program, and After-School Snacks and Meals. WIC and CSFP were combined as one option in the survey. These program variables were considered in selected previous studies associated with the EFNEP at state, regional, or national levels [19].

We excluded individuals who failed to answer any of the pertinent survey questions, those who chose not to report their gender, race, or public assistance program participation status, and those with implausible responses (such as reporting age to be zero or household size to be 128). We also excluded individuals who failed to complete dietary recalls for both the pre- and post-surveys, as these were necessary to calculate the HEI total score. After data screening, the study included **4,253** observations or participants. Out of 5,855 individuals surveyed, 405 did not provide race; 103 did not provide ethnicity; 1,191 did not provide income; 142 had age at zero; one individual provided a household size of 128; one individual did not provide participation status in WIC/CSFP; and 10 individuals failed to provide information to calculate their HEI score before and after the EFNEP intervention respectively. For 255 individuals, multiple unanswered questions were evident. Altogether, 1,602 unique individuals were removed from our analysis. The temporal and geographical distributions of these observations are presented in Table 1. Most counties experienced a peak in observations in FY2019 and FY2020, followed by a sharp decline in FY2021, perhaps partially explained by the COVID-19 pandemic.

**Table 1:** Number of observations by county and fiscal year.

Fiscal Year					
County	2019	2020	2021	2022	County Total
Bexar	176	221	78	72	547
Cameron	112	220	39	50	421
Dallas	334	144	12	23	513
El Paso	188	189	27	91	495
Harris	219	190	96	159	664
Hidalgo	253	206	113	281	853
Kleberg	19	0	0	0	19
Nueces	99	91	16	13	219
Tarrant	180	53	17	54	304
Travis	92	123	3	0	218
Year Total	1,672	1,437	401	743	4,253

**Fig 1:**
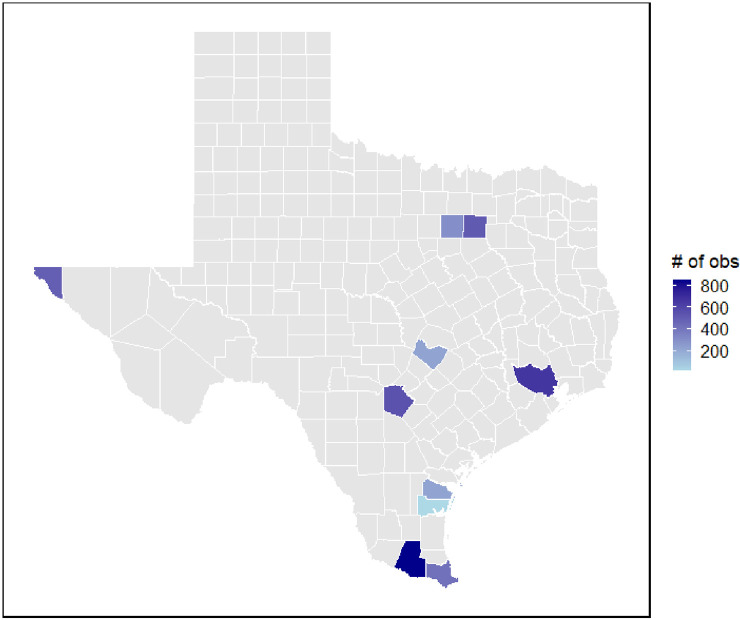
Geographical distribution of observations across ten Texas counties.

In Fig 1, we present a visualization of the total number of observations collected across the ten counties in Texas, with darker shades of blue indicating higher counts. The counties with the most observations primarily were associated with the southern and southeastern regions of the state, with clusters in Hidalgo, Cameron, and Bexar counties.

### Data analysis

All statistical analyses were conducted using R, version 4.3.1, [25]. Most previous studies employed a pre/post design [18], which we adopted in our analysis. Descriptive statistics, including the mean, median, standard deviation, minimum, and maximum, were calculated for HEI scores before and after the EFNEP intervention. The normality of HEI scores was assessed using the Jarque-Bera (JB) test, which evaluates skewness and kurtosis [26]. Although the JB test rejected normality due to the large sample size, visual inspection of the histogram did not indicate notable deviations from normality. Nevertheless, to account for non-normality, we relied on non-parametric tests.

A paired-sample Wilcoxon signed-rank test was used to compare HEI scores before and after the intervention. We also employed the Kruskal-Wallis test to evaluate differences in HEI score changes across groups such as fiscal year, county, race, and public assistance program participation. These tests enabled robust comparison without relying on parametric assumptions.

We include density plots, bar charts, and comparisons across counties, fiscal years, demographics, and public assistance program participation status, accompanied by non-parametric tests. The aim was to delineate key trends and differences, providing a clearer understanding of how various factors influence the change of the HEI before and after the EFNEP intervention.

**Fig 2:**
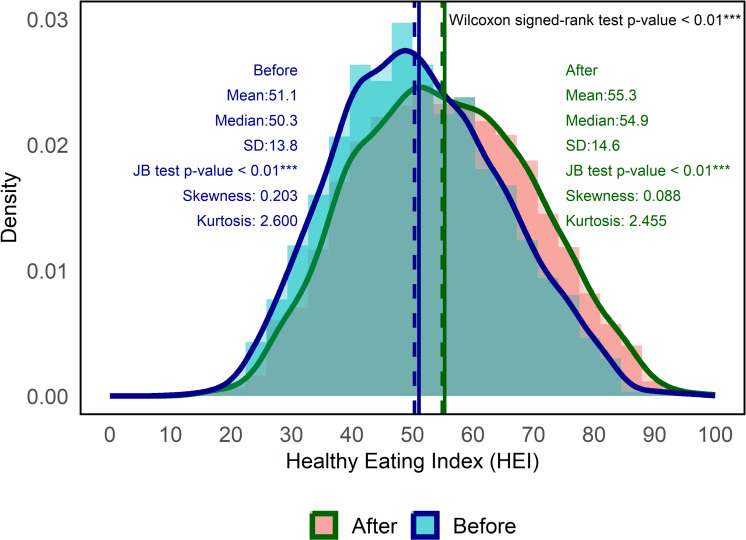
Density plot and summary statistics of healthy eating index (HEI) total score before and after intervention.

In Fig 2, we present density plots and descriptive statistics of the HEI sample scores pre- and post-intervention. The solid lines represent the mean HEI scores (51.1 before and 55.3 after the intervention), while the dashed lines indicate the median scores (50.3 before and 54.9 after the intervention). Based on the Wilcoxon signed-rank test, the 4.6 difference in median scores was deemed to be statistically different from zero at the 0.01 level. Hence, the EFNEP intervention provided a statistically significant improvement in HEI scores. In past studies, no clear distinction was made between statistically significant and clinically significant changes in HEI scores. While the benchmark for a clinically significant change in HEI score has been debated, Kirkpatrick [27] suggested this difference to be in the neighborhood of 5–6 points.

The standard deviation, skewness, kurtosis, and JB test p-values are provided for both distributions. The JB tests confirm that the HEI scores were not normally distributed (p-value < 0.01). The skewness metrics associated with the HEI scores before and after the intervention were 0.203 and 0.088, respectively, and the kurtosis metrics associated with the HEI scores before and after the intervention were 2.600 and 2.455, respectively. The skewness and kurtosis coefficients were different from those associated with a normal distribution (skewness coefficient of 0 and kurtosis coefficient of 3). The JB test is dependent not only on these measures but also on the sample size. Given the relatively large sample size coupled with the skewness and kurtosis metrics, normality of the HEI scores pre- and post-intervention was rejected.

In Table 2, we provide descriptive statistics for the change of the total HEI score, the total number of hours of the EFNEP education sessions, number of children, household size, age, and income. The mean change of HEI scores was 4.23. Notably, 1,689 participants (~40%) experienced a decline in the HEI score. Participants averaged slightly more than 9 hours taught in educational sessions. The average household size was between 4 and 5 members. The mean age of participants was approximately 40 years. All participants were from low-income households based on the federal poverty guideline. On average, participants’ annual income was $1,771, with a maximum of $25,000. The medians are all closely aligned with the mean values for these variables.

**Table 2:** Descriptive statistics for individuals’ healthy eating index (HEI) and other continuous variables.

Variable	Mean	25^th^ Percentile	Median	75^th^ Percentile	Minimum	Maximum
Change of HEI	4.23	-7.00	4.10	15.70	-67.00	63.5
Number of hours taught in EFNEP	9.19	7.50	9.00	11.00	1.00	19.50
Number of children	2.06	1.00	2.00	3.00	0.00	10.00
Household size	4.56	3.00	4.00	6.00	1.00	16.00
Age	40.04	31.00	38.00	47.00	16.00	93.00
Income/$1,000	1.77	0.76	1.50	2.40	0.00	25.00

In Table 3, we focus on binary demographic variables, while in Table 4, we examine participants’ involvement in other public assistance programs. Around 91% of the sample participants were white, 6% were black, and 1% were Asian. The other category concerning race includes individuals who reported to be American Indian, Alaska Native, Native Hawaiian or Pacific Islander. Individuals who reported themselves to be multiracial were also placed in this category. About 2% of the sample participants were characterized as these other races. Around 91% of the sample participants were female, and 87% were identified as Hispanic. According to Atoloye et al. [19], all previous studies associated with evaluations of EFNEP included women, but only a limited number of studies included men. Roughly 6% of the sample participants lived in rural areas, 3% were pregnant, 4% were nursing. Participation rates in SNAP, CNP, and WIC/CSFP were 30%, 59%, and 20%, respectively. Participation rates in Head Start, TANF, and TEFAP were 6%, 2%, and 4% respectively. Only 21 participants (~0.5%) were involved in FDPIR.

**Table 3:** Descriptive statistics associated with the binary demographic variables.

Variable	Proportion
Female	0.91
Male	0.09
Hispanic	0.87
Rural	0.06
White	0.91
Black	0.06
Asian	0.01
Other Races	0.02
Is pregnant	0.03
Is nursing	0.04

Note: Other Races incorporate multiracial, American Indian, Alaska Native, Native Hawaiian or Pacific Islander.

**Table 4:** Descriptive statistics associated with participation status in public assistance programs.

Variable	Proportion
SNAP	0.30
Child Nutrition Programs	0.59
FDPIR	0.01
Head Start	0.06
TANF	0.02
TEFAP	0.04
WIC/CSFP	0.20

Note: SNAP: Supplemental Nutrition Assistance Program; FDPIR: Food Distribution Program on Indian Reservations; TANF: Temporary Assistance for Needy Families; TEFAP: Emergency Food Assistance Program; WIC/CSFP: Special Supplemental Nutrition Program for Women, Infants, and Children (WIC) or Commodity Supplemental Food Program (CSFP).

**Fig 3:**
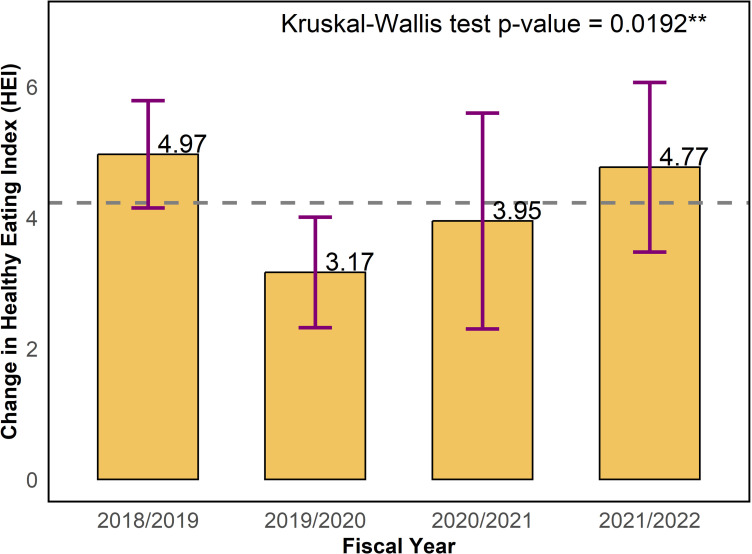
Change in healthy eating index (HEI) total score across fiscal years (2019-2022).

In Fig 3, we display a bar chart denoting the change in HEI total score across fiscal years. The change in HEI was not uniform across fiscal years based on the Kruskal-Wallis test (p = 0.0192). On average, the change in HEI across fiscal years ranged from 3.17 (FY2019/2020) to 4.97 (FY2018/2019). Statistically significant differences in the change in HEI score were identified for each of the four fiscal years.

**Fig 4:**
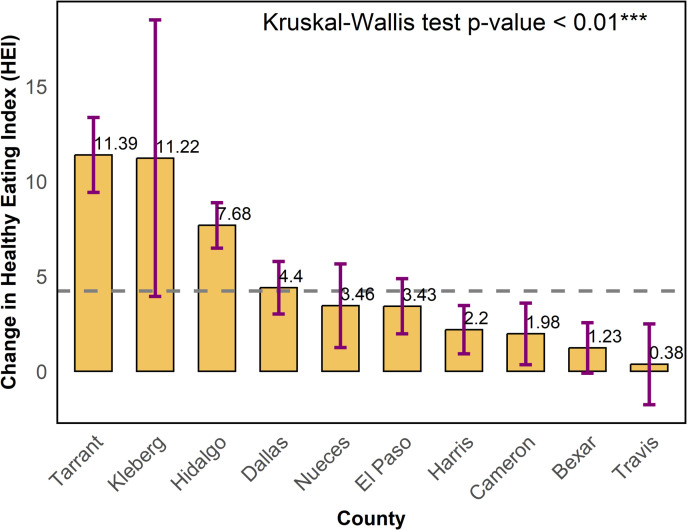
Change in healthy eating index (HEI) total score by counties.

In Fig 4, we provide a bar chart denoting the change in HEI total score by county. Geographically, statistically significant differences in the change in HEI were evident across counties based on the Kruskal-Wallis test (p < 0.01). On average, the change in HEI total score varied from 0.38 (Travis County) to 11.39 (Tarrant County). Dietary improvements attributed to EFNEP were most notable in Tarrant and Hildalgo Counties with changes in HEI of 11.39 and 7.68, respectively. In addition, a difference in HEI of 11.22 was evident in Kleberg County. That said, we acknowledge the limited number of EFNEP participants observed (n=17) in this county.

**Fig 5:**
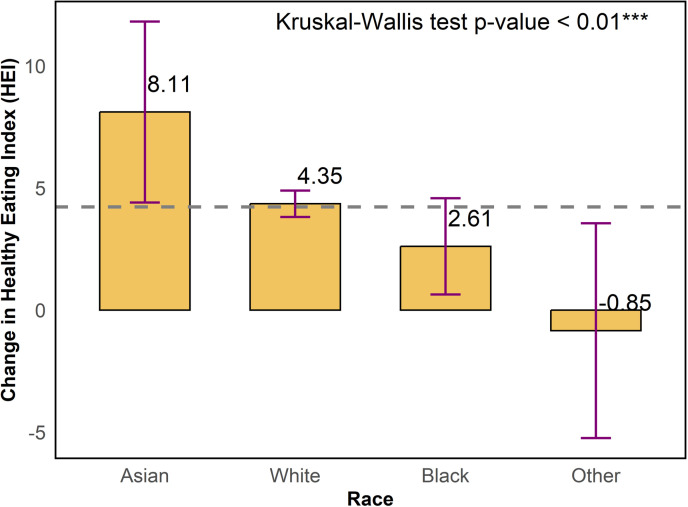
Change in healthy eating index (HEI) total score by races.

As shown in Fig 5, we provide a bar chart denoting the change in HEI total score by race. Concerning racial groups, participants identified as Asian showed the greatest post-intervention improvement in the change in HEI total score at 8.11 on average, followed by participants identified as white at 4.35 on average, participants identified as black at 2.61 on average, and participants identified as from other races at -0.85 on average. Statistically significant differences in the change in HEI total score were evident for Asians, whites, and blacks but not for other races. Differences among racial groups concerning the change in HEI total score were statistically significant based on the Kruskal-Wallis test (p < 0.01).

**Fig 6:**
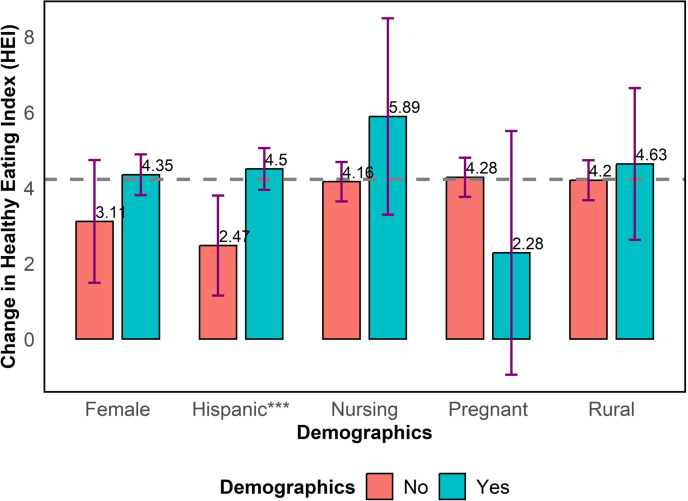
Change in healthy eating index (HEI) total score by selected demographic characteristics.

As exhibited in Fig 6, we provide the changes in HEI total score by selected demographic characteristics. Although the change in HEI total score was higher for females (4.35) compared to males (3.11), this difference was not statistically different from zero. Hispanic participants registered a statistically significant greater dietary improvement compared to non-Hispanic participants (4.50 vs. 2.47), as determined by the Wilcoxon signed-rank test (p < 0.01). No significant differences in the change in HEI total score were evident by nursing status (5.89 for participants who were nursing vs 4.16 for participants who were not nursing), pregnancy status (4.28 for participants who were not pregnant vs 2.28 for participants who were pregnant), or rural/non-rural classification (4.63 for participants from rural areas vs 4.20 for participants who were from non-rural areas).

**Fig 7:**
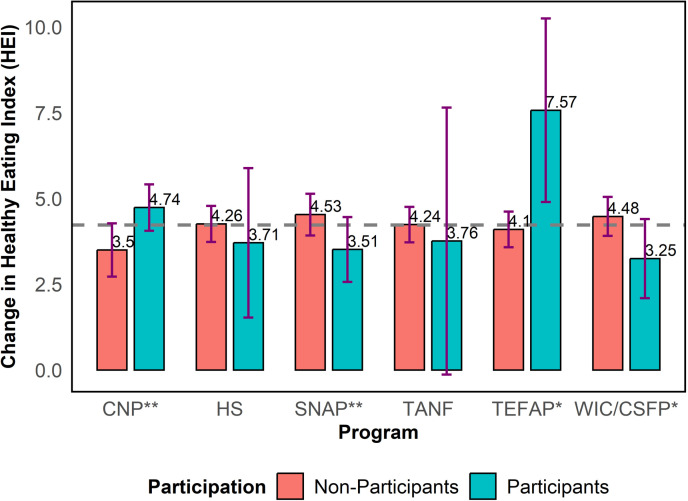
Change in healthy eating index (HEI) total score by participation status for selected public assistance programs.

As depicted in Fig 7, integration of the Texas EFNEP with several public assistance programs resulted in heterogeneous changes in HEI total scores, supported by Wilcoxon signed-rank tests (p-value = 0.0356 for CNP, p-value = 0.0481 for SNAP, p-value = 0.0391 for TEFAP, and p-value = 0.0638 for WIC/CSFP). Particularly, participants in CNP and TEFAP experienced higher increases in the change in HEI total scores of 4.74 and 7.57, on average, compared to 3.50 and 4.10, on average, for non-participants in these programs. On the other hand, participants in SNAP and WIC/CSFP experienced lower increases in the change in HEI total scores of 3.51 and 3.25, on average, compared to 4.53 and 4.48, on average, for non-participants in these programs. Although the changes in HEI total scores were lower for participants vs non-participants concerning the Head Start program and the TANF program, these differences were not statistically different from zero.

Finally, we explored the changes across all 13 component HEI categories. Descriptive statistics reported in Table 5 indicated statistically significant improvements in HEI scores for 12 out of 13 components. Notably, there were positive changes in the HEI scores for adequacy components, reflecting improved proportions in the diet of total fruit (0.61), whole fruit (0.62), total vegetables (0.18), greens and beans (0.27), whole grains (0.58), dairy (0.36), total protein foods (0.05), and seafood and plant protein (0.12). The significant improvements in these scores suggest that the EFNEP intervention in Texas effectively promoted a diet more aligned with DGA recommendations regarding these food groups. However, the component for fatty acids did not show a statistically significant positive change.

**Table 5:** Changes in healthy eating index (HEI) scores for all thirteen HEI categories.

Categories	Mean	25^th^ Percentile	Median	75^th^ Percentile	Minimum	Maximum	p-value
Total Fruit	0.61	–0.1	0	2.3	–5	5	<0.01
Whole Fruit	0.62	0.0	0	2.2	–5	5	<0.01
Total Vegetables	0.18	–0.8	0	1.4	–5	5	<0.01
Greens and Beans	0.27	0.0	0	0.4	–5	5	<0.01
Whole Grains	0.59	–0.6	0	2.6	–10	10	<0.01
Dairy	0.36	–2.7	0	3.5	–10	10	<0.01
Total Protein Foods	0.05	0.0	0	0	–5	5	0.03
Seafood and Plant Protein	0.12	–0.5	0	1.2	–5	5	<0.01
Fatty Acids	0.11	–3.0	0	3.3	–10	10	0.13
Refined Grains	0.80	–2.1	0	4.4	–10	10	<0.01
Sodium	-0.16	–3.0	0	2.6	–10	10	0.02
Added Sugar	0.41	–0.3	0	1.5	–10	10	<0.01
Saturated Fat	0.27	–2.5	0	3.2	–10	10	<0.01

*Note: p-values were derived from the use of the paired Wilcoxon signed-rank test.*

Concerning the moderation components, we observed statistically significant positive changes in refined grains (0.8), added sugar (0.41), and saturated fat (0.27). Thus, the EFNEP intervention in Texas was responsible for making the diet more aligned with DGA recommendations. Conversely, on average, the change in the HEI score for sodium was significantly higher by 0.16, indicating a deviation from DGA recommendations.

**Fig 8:**
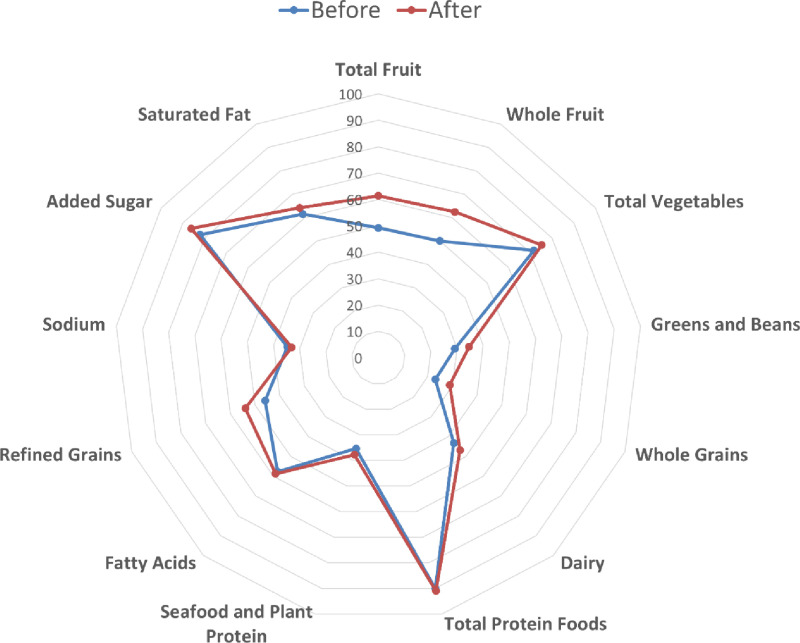
Radar plots for healthy eating index (HEI) scores for all thirteen HEI categories.

A radar plot exhibited in Fig 8 demonstrates that the “before” scores (blue line) were largely contained within the “after” scores (red line). This plot further illustrates that the intervention led to overall improvements across most components of the Healthy Eating Index (HEI). All scores were normalized on a scale of 0–100 for each category. Notably, the subcomponent for total protein foods showed only a minimal change between the pre- and post-intervention scores. This result was likely due to a do-called ceiling effect, as this component often starts near its maximum possible value, leaving limited room for improvement. While the change in total protein foods was statistically significant due to the large sample size, the small improvement in this category is unlikely to be clinically or practically significant.

### Regression analysis

To explore the relationships among various factors concerning their impact on the change in the HEI before and after the EFNEP intervention, we conducted a regression analysis. This analysis is crucial to address interactions among participation in selected public assistance programs, fiscal years, geographic location by county, and socio-economic demographic characteristics.

To account for these factors as well as to capture the spatial-temporal heterogeneity of the impact of EFNEP, we adopted a two-way fixed effects model given by:


$${\text{y}}_{\text{ijt}}={\text{x}}_{\text{ijt}}^{\prime }\beta +\theta_{j}+\mu_{t}+\varepsilon_{ijt},$$

where $y_{it}$ denotes the change of the HEI total score of participants living in county *i* surveyed in fiscal year *t*. ${\text{x}}_{\text{ijt}}$ consists of the vector of explanatory variables exhibited in Tables 2–4-4 for each individual *i* in year *t* in county *j* in addition to the intercept. *β* is the corresponding vector of the coefficients associated with ${\text{x}}_{\text{ijt}}$. $\theta_{j}$ and $\delta_{t}$ represent the county and year fixed effects, respectively, and $\varepsilon_{ijt}$ is the idiosyncratic error term. Reference categories delineated in Table 6 associated with the set of binary variables were excluded to avoid the dummy variable trap.

**Table 6:** Econometric results associated with the change in healthy eating index (HEI) total score.

Dependent Variable: Change in HEI Total Score			
Explanatory Variable	Coefficient	Standard Error	p-value
Female^a^	0.77	0.86	0.37
Hispanic^b^	1.39	1.05	0.19
Rural^c^	–0.77	1.04	0.46
Black^d^	0.94	1.35	0.49
Asian^d^	**6.82**	**2.13**	**<0.01**
Other races^d^	–2.90	2.19	0.18
Participant is Pregnant^e^	–1.35	1.86	0.47
Participant is Nursing^e^	**3.28**	**1.43**	**0.02**
Number of Children	–0.02	0.31	0.96
Household size	–0.17	0.22	0.44
Number of hours taught in EFNEP	–0.06	0.11	0.57
Age of Participant	–0.01	0.02	0.6
Participation in SNAP^f^	–0.74	0.62	0.24
Participation in CNP^f^	0.45	0.58	0.43
Participation in FDPIR^f^	–2.14	3.61	0.55
Participation in Head Start^f^	0.04	1.18	0.97
Participation in Other Programs^f^	–0.65	1.06	0.54
Participation in TANF^f^	–0.84	2.1	0.69
Participation in TEFAP^f^	2.18	1.46	0.14
Participation in WIC/CSFP^f^	–0.86	0.73	0.24
Income/$1,000	–0.14	0.16	0.37
Cameron^g^	0.80	1.13	0.48
Dallas^g^	**2.25**	**1.1**	**0.04**
El Paso^g^	1.78	1.04	0.09
Harris^g^	0.55	1.01	0.58
Hidalgo^g^	**6.06**	**1.03**	**<0.01**
Kleberg^g^	**12.35**	**4.39**	**<0.01**
Nueces^g^	1.47	1.39	0.29
Tarrant^g^	**8.89**	**1.21**	**<0.01**
Travis^g^	–1.80	1.33	0.18
Fiscal year 2019/2020^h^	–1.10	0.61	0.07
Fiscal year 2020/2021^h^	–0.71	0.98	0.47
Fiscal year 2021/2022^h^	–1.06	0.77	0.17
Influential observation #1	**46.15**	**20.57**	**0.02**
Influential observation #2	**39.63**	**17.41**	**0.02**
Influential observation #3	–**38.99**	**14.2**	**<0.01**
Influential observation #4	–**40.08**	**13.94**	**<0.01**
Influential observation #5	–**34.43**	**16.31**	**0.03**
Influential observation #6	–**36.41**	**14.02**	**<0.01**
Influential observation #7	**46.58**	**19.1**	**0.01**
Influential observation #8	**51.61**	**21.82**	**0.02**
Influential observation #9	–**33.49**	**17.28**	**0.05**
Influential observation #10	**33.68**	**14.83**	**0.02**
Influential observation #11	–**46.29**	**16.96**	**<0.01**
Influential observation #12	**42.93**	**19.28**	**0.03**
Influential observation #13	**32.06**	**14.52**	**0.03**
Influential observation #14	**34.77**	**13.47**	**<0.01**
Influential observation #15	–**31.57**	**13.95**	**0.02**
Influential observation #16	**31.62**	**13.58**	**0.02**
Influential observation #17	–**32.96**	**14.27**	**0.02**
Constant	2.51	2.03	0.22
# Observations		4,253	
R^2^		0.06	
Adjusted R^2^		0.05	
Standard error of the regression (SER)		1.881 (df=4,202)	
F Statistic		**5.47** **(df=50; 4,208)**	**<0.01**

^a^ *reference category--male;*

^b^ *reference category--non-Hispanic;*

^c^ *reference category--non-rural.;*

^d^ *reference category--white;*

^e^ *reference category—participant is not pregnant or not nursing;*

^f^ *reference category—non-participation in the public assistance program in question;*

^g^ *reference category—Bexar County;*

^h^*reference category-fiscal year 2018/2019*; *influential observation is defined as any observation that is a leverage point and an outlier [*29*].*

Note: SNAP: Supplemental Nutrition Assistance Program; CNP: Child Nutrition Programs; FDPIR: Food Distribution Program on Indian Reservations; TANF: Temporary Assistance for Needy Families; TEFAP: Emergency Food Assistance Program; WIC/CSFP: Special Supplemental Nutrition Program for Women, Infants, and Children (WIC) or Commodity Supplemental Food Program (CSFP). The fiscal year is defined as the 12-month period beginning on October 1 of the previous year and ending on September 30 of the following year. Estimated coefficients associated with bolded text are statistically different from zero at the 0.05 level.

Source: Estimation by the authors using R, version 4.3.1.

To mitigate any issues attributed to heteroscedasticity, defined as unequal variances of the error terms, particularly biased standard errors of the estimated regression coefficients, we utilized weighted least squares (WLS) as the estimation procedure in lieu of ordinary least squares (OLS) as the estimation procedure. The use of weighted least squares (WLS) entails a three-stage procedure. In the first stage, we estimated the model using OLS to obtain the corresponding OLS residuals. Next, we ran a supplementary regression on the logarithm of the square of the OLS residuals, using the same explanatory variables in the model specification, to detect the presence/non-presence of heteroscedasticity. The supplementary regression is congruent to the Harvey test of heteroscedasticity [28]. Because we rejected the null hypothesis of the absence of heteroscedasticity based on the Harvey test, we calculated the exponentiated inverses of the fitted values from this auxiliary regression to be used as weights in a subsequent regression to complete the WLS estimation.

Based on variance inflation factors, condition indices, and variance decomposition proportions [29], no evidence of degrading collinearity was detected. Based on the R-student statistics and hat-diagonal elements [29], 17 influential observations, defined as both outliers and leverage points, were detected. To circumvent the issues associated with these influential observations, separate dummy variables were generated for each of these observations and included in the model specification. In the regression analysis using WLS as well as capturing influential observations, the goodness-of-fit metric (R^2^) was 0.06. The magnitude of this measure is consistent with past studies that use repeated cross-sectional data in regression analysis. The standard error of the regression was estimated to be 1.881.

In Table 6, we present the estimation results associated with the various explanatory variables concerning the change in HEI total score, utilizing the WLS procedure adjusting for influential observations. The regression results substantiated the previously discussed findings from the data analysis based on the use of non-parametric tests. The regression analysis however considers all explanatory factors simultaneously, whereas the non-parametric analyses do not.

Relative to participants identified as white, the change of HEI was significantly lower by 6.82 for participants identified as Asian. Additionally, the change of HEI was higher by 3.28 for individuals who were nursing compared to those who were not.

The change of HEI was higher for participants residing in Dallas, Hidalgo, Kleberg, and Tarrant Counties by 2.25, 6.06, 11.35, and 8.89, respectively, compared to participants residing in Bexar County, all else being equal.

Based on the regression analysis, no statistically significant differences in the percentage change of the HEI were evident by fiscal year. Additionally, household size, income, age, gender, pregnancy status, ethnicity, rural/urban residence, number of hours taught in EFNEP, or the participation status of other social welfare programs were not statistically significant factors affecting the change of HEI.

## Discussion

Contributing to the body of literature aimed at understanding the effectiveness of nutrition education interventions, we sought to identify if the Texas EFNEP led to improvements in overall dietary quality as measured by the change in the total HEI score. The strengths of this study were the large sample size over four recent fiscal years from ten heterogeneous Texas counties as well as the use of HEI scores based on the 2015 Dietary Guidelines. Moreover, we considered a diverse set of socio-demographic variables as well as participation status in public assistance programs.

The mean change in 2015 HEI scores was 4.23, indicative that the EFNEP in Texas was indeed effective in improving the overall quality of diets of participants, at least in the short term. The change in the HEI was not uniform across fiscal years, ranging from 3.17 (FY2019/2020) to 4.97 (FY2018/2019). The dip in the change of HEI for FY2019/2020 was likely related to the onset of the COVID-19 pandemic, which likely disrupted participants’ access to food, nutrition education. Social distancing measures also may have limited program delivery or participants’ ability to make healthier food choices during the pandemic.

Geographically, dietary improvements attributed to EFNEP in Texas were most notable in Tarrant County and Hildalgo County with changes in the HEI of 11.39 and 7.68, respectively. Hidalgo County is known for its high rates of poverty and food insecurity. The pre-EFNEP HEI score for participants in Hidalgo County was 49.8, the third lowest among ten counties considered in this study. The statistically significant improvement in HEI suggests that the Texas EFNEP is effectively reaching and benefiting households living in this county who may have had greater potential for dietary improvement due to pre-existing poor diet quality. On the other hand, Tarrant County includes urban areas like Fort Worth, where EFNEP has been implemented through partnerships with local agencies such as AVANCE, Blue Zones, Boys and Girls Clubs of Greater Fort Worth, Child Care Associates, and the Fort Worth Independent School District. These collaborations have likely enhanced the program’s outreach efforts and contributed to the observed improvements in dietary quality among participants.

Among racial groups, participants identified as Asian showed the greatest post-intervention improvement in HEI scores at 8.11 on average, followed by participants identified as white at 4.35 on average, and participants identified as black at 2.61 on average. Hispanic participants registered a statistically significant greater dietary improvement compared to non-Hispanic participants (4.50 vs. 2.47). The relatively higher improvement in the total HEI score among Asian and Hispanic participants suggests that the program successfully aligned with dietary preferences or health motivations within these ethnic and racial groups. Conversely, the relatively lower HEI improvements among non-Hispanic, black, and other race participants indicates that these groups require more culturally relevant educational materials or different strategies to engage them effectively. In the Texas EFNEP, we recommend developing more targeted interventions that account for cultural differences in diet, food preparation, and health perceptions.

No significant differences in the change in HEI were evident by gender, nursing status, pregnancy status, or rural/non-rural classification. Integration of the Texas EFNEP with specific public assistance programs, namely CNP and TEFAP, resulted in enhanced changes in total HEI scores.

For the most part, the regression results substantiated the previously discussed findings based on the use of non-parametric tests. Based on the regression analysis, nursing status was a key determinant associated with the change in the HEI total score. However, household size, income, age, gender, pregnancy status, rural/urban residence, and number of hours taught in EFNEP were not statistically significant factors affecting the change in the HEI total score. In the future, other ways of teaching or presenting lessons to EFNEP participants should be considered in Texas given the non-significance of the number of hours taught.

Statistically significant differences in the change in HEI scores were positive for eight out of nine adequacy components, namely total fruit, total vegetables, greens and beans, whole grains, dairy, total protein foods, and seafood and plant protein. The exception was for fatty acids. Perhaps participants were not able to adjust their intake toward healthy fats, such as omega-3 and omega-6 fatty acids. On average, increases in HEI scores for refined grains, added sugar, and saturated fat reflected significantly greater alignments with DGA recommendations regarding these components.

Conversely, the EFNEP intervention was ineffective in aligning participants’ sodium intakes with DGA recommendations. Foods that are inexpensive and accessible to lower-income populations such as canned soups, frozen meals, snacks, and fast foods are high in sodium. Further, participants may have limited time or resources to prepare meals from scratch and hence rely on pre-prepared or restaurant foods where they have less control over sodium content [30,31]. Moreover, measuring individual-level changes in sodium consumption is inherently challenging, as sodium is a micronutrient whose intake is difficult to assess accurately through self-reported dietary recalls. The gold standard for assessing sodium intake involves multiple 24-hour urine collections [32], which was not feasible in this study. This limitation in measurement may partially explain why there was no evident effect on the sodium subcomponent score due to EFNEP.

The information gleaned from this analysis is useful to policy stakeholders associated with the Texas Department of Agriculture and the U.S. Department of Agriculture. The bottom-line takeaway from this study is that the Texas EFNEP contributed to statistically significant improvements in overall dietary quality, as reflected in the change in total HEI score and the change HEI score for adequacy and moderation components. However, the mean change in HEI scores of 4.23 represents a modest improvement that may not reach the threshold for clinical significance. Furthermore, while approximately 40% of participants experienced a decline in their HEI score, this finding likely reflects variability in self-reported dietary recalls rather than a clinically significant deterioration in diet quality for most participants. Generally speaking, the findings from our study in Texas align with previous studies associated with the effectiveness of EFNEP conducted in other states, regions, or nationally.

### Limitations

As pointed out by Chipman and Kendall [1], the tools used in evaluating the success of EFNEP have limitations. The 24-hour food recall or knowledge and behavior questionnaires may not adequately reflect program effectiveness. In addition, this dataset did not measure or assess if improvements were maintained post-graduation. Atoloye et al. [19] identified the need to measure long-term outcomes. Further, our analysis did not consider education level because the Texas EFNEP excluded this demographic characteristic from consideration. Finally, no attention was paid to food insecurity in this analysis.

Additionally, we acknowledge another limitation inherent in dietary intervention studies, namely that participants may misreport their dietary intake over time. As noted by Thompson et al. [33], self-reported dietary data can be subject to recall bias, social desirability bias, or errors in portion size estimation. These factors could potentially affect the accuracy of the 24-hour dietary recalls collected before and after the intervention, leading to over- or under-estimation of dietary changes. Future studies could mitigate this issue by incorporating objective measures of dietary intake, such as biomarkers, to complement self-reported data.

## Implications for research and practice

Without question, this study provided encouraging results associated with improving overall dietary quality of Texas participants. The results gleaned from this study are beneficial to practitioners, cooperative extension professionals, and nutrition education researchers. Findings highlight the impact that nutrition education programs like EFNEP can have on low-income families in Texas. Importantly, in any analysis dealing with ascertaining the effectiveness of EFNEP, it is paramount to consider other federally funded public assistance programs.

Future work is needed to expand this work to generalize the results to other geographical counties in Texas and to more current fiscal years. In subsequent studies, consideration also should be given to education level and food insecurity as well as behavioral questions related to: (1) planning meals before grocery shopping; (2) comparing prices when shopping; (3) using a grocery list when shopping for food; (4) thinking about healthy food choices; (5) using the information presented on food labels; and (6) budgeting enough money for food. Finally, longitudinal studies are needed to conclude that participating in the Texas EFNEP results in sustained dietary improvement.
